# Association of waist-to-height ratio with all-cause and obesity-related mortality in adults: a prospective cohort study

**DOI:** 10.3389/fnut.2025.1614347

**Published:** 2025-08-11

**Authors:** Gang Wang, Yunpeng Luo, Tianyi Yang, Jukai Huang, Jiaoyue Li, Yan Liu, Xiaohui Yang

**Affiliations:** ^1^Dongzhimen Hospital, Beijing University of Chinese Medicine, Beijing, China; ^2^Institute for Health, Health Care Policy and Aging Research, Rutgers University, New Brunswick, NJ, United States

**Keywords:** waist-to-height ratio, visceral fat, mortality, obesity, cohort study

## Abstract

**Background:**

The waist-to-height ratio (WHtR) is the optimal indicator for assessing obesity-related diseases. Establishing a unified standard for investigating the relationship between WHtR and mortality is an urgent need.

**Methods:**

This cohort study included 47,741 U. S. adults from the National Health and Nutrition Examination Survey database from 1999 to 2018. The survival outcomes were all-cause mortality and obesity-related mortality. The associations between WHtR and mortality were quantified using restricted cubic splines and Cox proportional hazards regression models.

**Results:**

Among the 47,741 participants, the association between WHtR and all-cause mortality was characterized by a distinct U-shaped curve, with an inflection point at 0.58. The relative risk was minimized in the Q3 category, with a hazard ratio of 0.753 (95% CI, 0.752–0.754). WHtR demonstrated a J-shaped nonlinear relationship with the risk of mortality from cardiovascular disease, cancer, and diabetes (*p* < 0.001), with an inflection point of 0.58 for each condition. A higher WHtR (≥0.58) was associated with increased risks of mortality from cardiovascular disease (35.5%), cancer (4.5%), cerebrovascular disease (10.0%), and diabetes (69.8%). In subgroup analyses, the cutoff value of 0.58 for WHtR showed good stability across different populations.

**Conclusion:**

We found that the WHtR is associated with all-cause mortality in a U-shaped manner and provides a relatively stable cutoff value (0.58) for mortality related to obesity-associated diseases. This finding offers a convenient anthropometric indicator for body management in the general population.

## 1 Introduction

World Health Organization (WHO) investigations reveal a global weighted prevalence of overweight and obesity reaching 37.0%, with higher rates observed in high-income countries (1) Recent projections indicate a persistent rise in obesity rates among the U. S. population, accompanied by an escalating burden of obesity-related comorbidities including type 2 diabetes, cardiovascular diseases, malignancies, and stroke (2), collectively imposing substantial economic and public health challenges. The complex pathogenesis of these chronic conditions has hindered pharmacological development (3), with current therapies primarily delaying disease progression rather than reversing established pathology. However, accumulating evidence supports weight reduction as a therapeutic strategy capable of ameliorating or even reversing disease trajectories in these conditions (4), underscoring the critical need for anthropometrically guided weight management objectives.

It should be noted that traditional obesity indicators, such as BMI, waist circumference, and waist-to-hip ratio, are increasingly unable to meet clinical needs. On the one hand, traditional indicators are associated with an obesity paradox in the clinical outcomes of obesity-related chronic diseases (5), possibly due to the anti-inflammatory and cardioprotective effects of subcutaneous fat, while visceral fat is more harmful (6). This means that central obesity has a more significant impact on health than general obesity at the same BMI (7). On the other hand, the obesity cutoff values for traditional anthropometric indicators are greatly influenced by age, race, and gender, which limits their clinical application.

Contemporary research corroborates these limitations, advocating for supplementary anthropometric measures alongside BMI in clinical obesity assessment (8). The waist-to-height ratio (WHtR) has emerged as a pragmatic diagnostic tool, now incorporated into the European Association for the Study of Obesity’s revised criteria for central adiposity (9). WHtR demonstrates enhanced capacity to account for gender and ethnic variations in body composition while reliably reflecting visceral fat accumulation. A cohort study of 8,339 patients revealed that WHtR-based assessments more accurately predict cardiovascular hospitalization and mortality than conventional metrics, potentially resolving the obesity paradox (10). A multinational meta-analysis encompassing over 300,000 participants further established WHtR’s superior screening performance for diabetes, hypertension, cardiovascular disease, and metabolic syndrome compared to traditional indices (11). Despite these advantages, the absence of standardized WHtR thresholds remains a critical limitation. Recent investigations have proposed population-specific WHtR cutoffs. A multinational study of 24,000 adolescents (aged 6–18 years) from 10 countries determined optimal cutoffs: 0.46 for Asian/African youth versus 0.50 for European/North American youth (12). Furthermore, a Chinese cohort study on cardiovascular and metabolic diseases established sex-specific thresholds: 0.51 for males and 0.53 for females (13). Some previous studies considered a healthy WHtR to be less than 0.5, meaning the waist circumference should be less than half of the height (9, 14, 15), but this lacks sufficient validation. There’s an urgent need to establish a unified WHtR standard across different populations and diseases.

To our knowledge, large-scale investigations examining WHtR’s association with all-cause and obesity-attributable mortality remain scarce. This study leverages NHANES data to analyze relationships between WHtR and mortality from all causes and obesity-related diseases (type 2 diabetes, cardiovascular diseases, cancer, stroke), aiming to inform evidence-based anthropometric standards for clinical obesity management.

## 2 Methods

This project was approved by the Ethics Review Board (ERB) of the National Center for Health Statistics (NCHS), with approval numbers: Protocol #98-12, Protocol #2005-06, Protocol #2011-17, and Protocol #2018-01. The National Health and Nutrition Examination Survey (NHANES) was approved by the Institutional Review Board of the National Center for Health Statistics, and all participants provided written informed consent. This study adhered to the guidelines for reporting observational studies in epidemiology (STROBE).

### 2.1 Study participants

**Figure 1:**
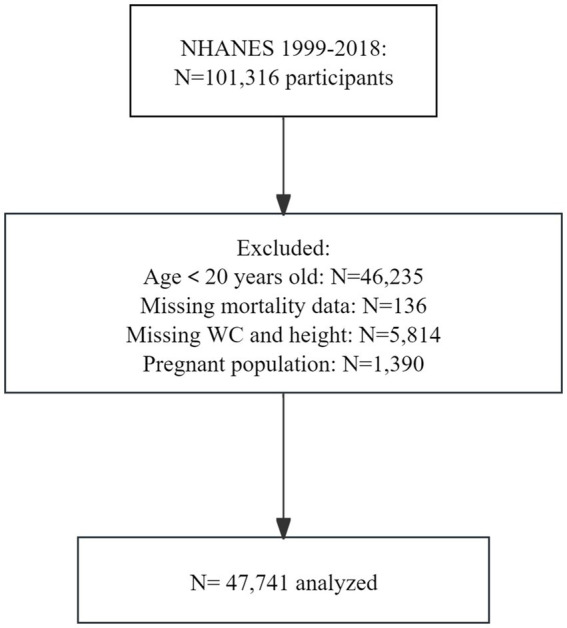
Flowchart of participant selection. WC, waist circumference.

All data have been publicly provided by the National Center for Health Statistics. Data from NHANES were utilized, which is a series of nationally representative cohort surveys designed to monitor the public health status of the U. S. population. Since 1999, NHANES has been conducted in 2-year cycles, collecting data from household interviews and examination visits in a mobile examination center. We included participants from ten NHANES cycles from 1999 to 2018, who were weighted to represent the non-institutionalized U. S. civilian population. Among the 101,316 respondents who fully met the eligibility criteria for analysis, 46,235 were excluded for being under 20 years of age, 136 were excluded due to missing mortality data, 5,814 were excluded for incomplete data on WHtR, and 1,390 were excluded due to pregnancy. The final sample size consisted of 47,741 adults (Figure 1).

### 2.2 Survival outcome

The outcome of interest was all-cause mortality and cause-specific mortality. Mortality data were obtained from the Centers for Disease Control and Prevention (CDC) website and linked to the NHANES database using unique subject identifiers, with death information ascertained up to December 31, 2019. Causes of death were defined according to the International Classification of Diseases and Related Health Problems, Tenth Revision (ICD-10) codes. Study participants were followed from the date of their survey participation until the date of death or the end of follow-up, whichever occurred first.

### 2.3 Waist-to-height ratio definition

According to the definition of adult obesity by the European Association for the Study of Obesity (EASO), the WHtR was calculated as the ratio of waist circumference to height (9). Given the lack of reference ranges for WHtR, we categorized WHtR into five groups based on the 20th, 40th, 60th, and 80th percentiles to explore its association with mortality. Based on the RCS results, we used 0.58 as the cutoff to split the WHtR into two groups, with those below 0.58 as the reference group, to assess stability across different influencing factors.

### 2.4 Covariates

Information on age, sex, race and ethnicity, education level, and poverty-income ratio (PIR; the ratio of family income to the poverty threshold, with higher ratios indicating higher income levels) was collected during household interviews. Smoking status, alcohol consumption status, family history of cardiovascular disease (CVD), and family history of diabetes were also collected. Participants who had never smoked or had smoked fewer than 100 cigarettes in their lifetime were defined as never - smokers. Current smokers were those who had smoked 100 or more cigarettes in their lifetime and were still smoking. Former smokers were those who had smoked 100 or more cigarettes in their lifetime but had quit smoking completely. Drinkers were defined as men who consumed more than 21 standard drinks per week and women who consumed more than 14 standard drinks per week. Race and ethnicity were self-reported by study participants based on fixed categories, including Mexican American, non-Hispanic Black people, non-Hispanic White people and other races and ethnicities (American Indian or Alaska Native, Native Hawaiian or Pacific Islander, and non-Hispanic Asian). Education level was categorized as less than 9th grade, 9th to 11th grade, high school graduate, some college, and college graduate or above.

### 2.5 Statistical analysis

All analyses accounted for the complex survey design of NHANES, incorporating survey weights to generate nationally representative estimates for the non-institutionalized U. S. civilian population. Continuous variables are presented as mean ± standard deviation, while categorical variables are summarized as frequencies and percentages. Between-group comparisons utilized Student’s t-tests or chi-square tests for normally distributed variables, with nonparametric alternatives applied to skewed distributions. Additionally, to ensure comparability among different variables, we standardized some variables.

To test non-linear relationships and determine the optimal WHtR cutoff for assessing all-cause and obesity-related disease mortality, we presented restricted cubic spline (RCS) curves with three knots. Then, we tested the Cox model proportional hazards assumption for the relationship between WHtR and mortality. We quantified the weighted association between WHtR and mortality using hazard ratios (HRs) and 95% confidence intervals (CIs), analyzing before and after adjusting for confounders. Additionally, we performed subgroup analyses on the study population across confounder strata.

We performed five iterations of multiple imputation to address missing data, complemented by sensitivity analyses to assess robustness. The imputation model assumed that the data were Missing At Random (MAR), and included all analysis variables plus auxiliary variables to predict missingness. To further strengthen our findings, we excluded individuals with less than 2 years of follow-up or who died from accidents for sensitivity analysis. Moreover, we analyzed the association between waist-to-height ratio and mortality in different age, sex, and race groups.

All analyses were performed using R programming version 4.4.2 (R Project for Statistical Computing). *p*-values were two-sided, with *p* < 0.05 considered statistically significant. The data analysis period was from December 1, 2024, to February 10, 2025.

## 3 Results

### 3.1 Baseline characteristics

**Figure 2:**
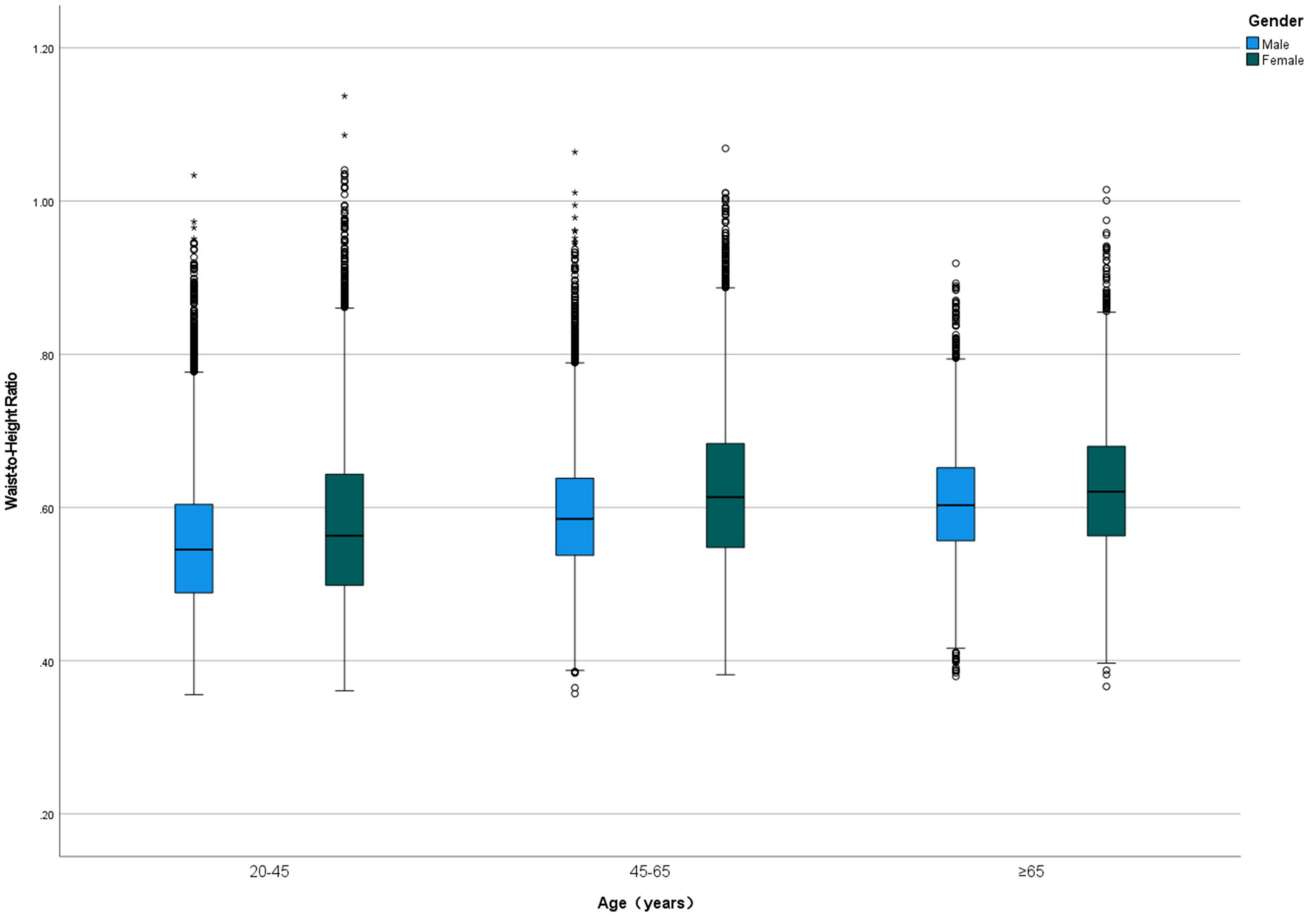
Comparison of waist-to-height ratio across different age groups and sexes.

Among 47,741 eligible adults with complete WHtR and mortality data, the mean age was 46.74 ± 17.86 years, with 23,994 (50.30%) being female. The cohort included 17.4% Mexican Americans, 21.0% non-Hispanic Black people, 44.1% non-Hispanic White people, and 17.5% individuals of other racial or ethnic groups. During a median follow-up period of 9.16 (5.00–13.91) years, 7,151 (15.0%) participants died, including 4,149 (58.0%) obesity-related deaths. WHtR values ranged from 0.36 to 1.14, with a mean of 0.59 ± 0.09. WHtR increased with age and was significantly higher in women (0.603 ± 0.102) compared to men (0.578 ± 0.087; *p* < 0.001), with females exhibiting higher WHtR values across all age groups (Figure 2). Participants were divided into quintiles based on WHtR (from quartile 1 to quartile 5), and their baseline characteristics are presented in Table 1.

**Table 1:** Baseline characteristics of the US adults in the national health and nutrition examination survey, 1999 to 2018 (*n* = 47,741).

Characteristic	Waist-to-height ratio					*P-*value
	Q1 (*n* = 8,351)	Q2 (*n* = 8,692)	Q3 (*n* = 10,227)	Q4 (*n* = 10,032)	Q5 (*n* = 10,439)	
Age						<0.001
20 to <45	5,809 (69.6)	4,421 (50.9)	4,047 (39.6)	3,270 (32.6)	3,396 (32.5)	
45 to <65	1,781 (21.3)	2,746 (31.6)	3,657 (35.8)	3,725 (37.1)	4,093 (39.2)	
≥65	761 (9.1)	1,525 (17.5)	2,523 (24.7)	3,037 (30.3)	2,950 (28.3)	
Sex						<0.001
Male	4,361 (52.2)	4,762 (54.8)	5,795 (56.7)	5,033 (50.2)	3,796 (36.4)	
Female	3,990 (47.8)	3,930 (45.2)	4,432 (43.3)	4,999 (49.8)	6,643 (63.6)	
Race and ethnicity						<0.001
Mexican American	741 (8.9)	1,338 (15.4)	2,015 (19.7)	2,161 (21.5)	2,075 (19.9)	
Non-Hispanic White people	3,960 (47.4)	3,887 (44.7)	4,423 (43.2)	4,276 (42.6)	4,485 (43.0)	
Non-Hispanic Black people	2,032 (24.3)	1,686 (19.4)	1,861 (18.2)	1,957 (19.5)	2,479 (23.7)	
Other race	1,618 (19.4)	1,781 (20.5)	1,928 (18.9)	1,638 (16.3)	1,400 (13.4)	
Education						<0.001
<9th Grade	447 (5.4)	797 (9.2)	1,367 (13.4)	1,591 (15.9)	1,517 (14.5)	
9th–11th Grade	1,127 (13.5)	1,161 (13.4)	1,567 (15.3)	1,514 (15.1)	1,721 (16.5)	
High school graduate	1,826 (21.9)	1,913 (22.0)	2,327 (22.8)	2,408 (24.0)	2,610 (25.0)	
Some college	2,498 (30.0)	2,412 (27.8)	2,758 (27.0)	2,752 (27.5)	3,099 (29.7)	
≥College graduate	2,442 (29.3)	2,400 (27.6)	2,197 (21.5)	1,754 (17.5)	1,486 (14.2)	
PIR						<0.001
<1	1,677 (20.7)	1,441 (17.2)	1,795 (18.3)	1,904 (19.9)	2,322 (23.3)	
≥1	6,430 (79.3)	6,932 (82.8)	8,037 (81.7)	7,683 (80.1)	7,642 (76.7)	
Cigarette smoking						<0.001
Never	4,600 (55.2)	4,771 (54.9)	5,418 (53.0)	5,283 (52.7)	5,647 (54.1)	
Former	1,242 (14.9)	1,926 (22.2)	2,708 (26.5)	2,940 (29.3)	2,960 (28.4)	
Current	2,498 (30.0)	1,988 (22.9)	2,097 (20.5)	1,797 (17.9)	1,827 (17.5)	
Alcohol drinking	2,517 (31.0)	2,374 (28.4)	2,830 (28.8)	2,772 (28.9)	2,915 (29.6)	0.35
Family history of CVD	834 (10.3)	921 (10.9)	1,151 (11.5)	1,278 (13.1)	1,666 (16.4)	<0.001
Family history of diabetes	2,788 (34.0)	3,365 (39.5)	4,305 (42.8)	4,679 (47.7)	5,620 (54.9)	<0.001

CVD, cardiovascular disease; PIR, poverty impact ratio.

### 3.2 WHtR and all-cause mortality

**Figure 3:**
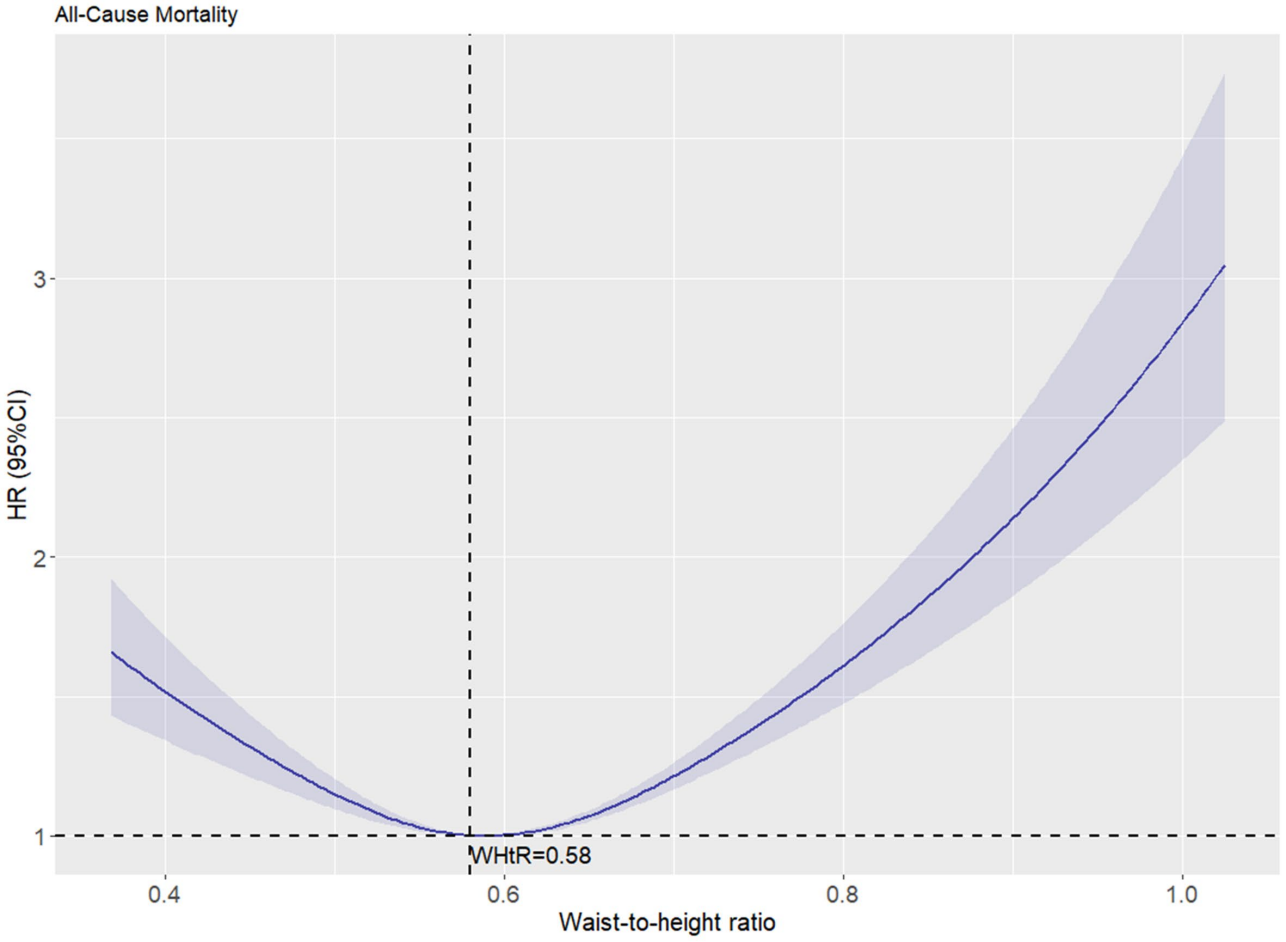
The association between waist-to-height ratio and all-cause mortality after adjustment. The solid curved line represents the estimates for the association of WHtR with mortality, and shading, the 95% CI.

Restricted cubic spline (RCS) analysis revealed a nonlinear relationship (*p* < 0.001) between WHtR and all-cause mortality after adjusting for confounders, demonstrating a distinct U-shaped curve with an inflection point at 0.58 (Figure 3). When WHtR was categorized into quintiles (Q1, lowest to Q5, highest), Cox regression quantified the association between WHtR and all-cause mortality. In the unadjusted model, WHtR showed a positive correlation with all-cause mortality (*p* < 0.001), with Q5 exhibiting a hazard ratio (HR) of 2.966 (95% CI, 2.961–2.970) compared to Q1. After adjusting for relevant confounders, the relative HR trend aligned with the RCS plot, showing an initial increase followed by a decrease. Using Q1 as the reference group, the relative HR reached its minimum value of 0.753 (0.752–0.754) in the Q3 range, while the HR in Q5 (≥0.66) was 1.112 (1.110–1.114). When WHtR ≥ 0.58, the HR increased by 11.2% (Table 2).

**Table 2:** Adjusted HR (95%CI) of waist-to-height ratio with all-cause mortality.

All cause mortality	Crude model^a^	Adjusted model^b^	*P-*value
Per 0.1	1.423 (1.423–1.424)	1.124 (1.123–1.124)	<0.001
Q1 (<0.50)	1 (Reference)	1 (Reference)	
Q2 (0.50–0.55)	1.470 (1.468–1.473)	0.847 (0.845–0.848)	<0.001
Q3 (0.55–0.60)	1.785 (1.782–1.788)	0.753 (0.752–0.754)	<0.001
Q3 (0.60–0.66)	2.593 (2.589–2.597)	0.887 (0.886–0.889)	<0.001
Q5 (≥0.66)	2.966 (2.961–2.970)	1.112 (1.110–1.114)	<0.001
WhtR ≥ 0.58	1.950 (1.948–1.951)	1.112 (1.111–1.113)	<0.001

HR, hazard ratio; Q, quantile.

a unadjusted.

b Adjusted for age, sex, race and ethnicity, education, income, smoking, drinking, family history of cardiovascular disease, and family history of diabetes.

### 3.3 WHtR and obesity-related disease mortality

**Figure 4:**
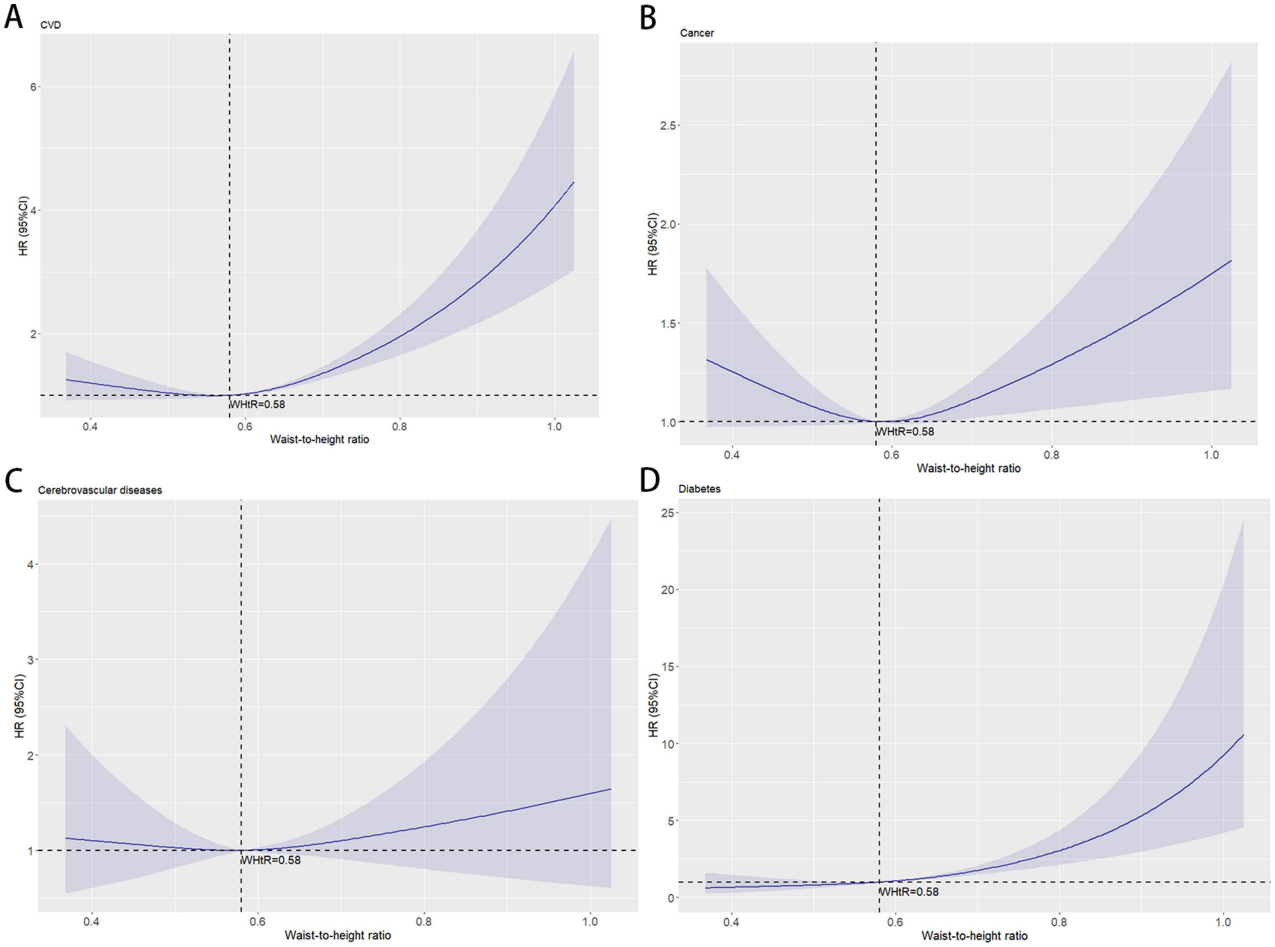
The association between waist-to-height ratio and obesity-related mortality after adjustment. The solid curved line represents the estimates for the association of WHtR with mortality, and shading, the 95% CI. **(A)** Association between waist-to-height ratio and cardiovascular diseases mortality; **(B)** Association between waist-to-height ratio and cancer mortality; **(C)** Association between waist-to-height ratio and cerebrovascular diseases mortality; **(D)** Association between waist-to-height ratio and diabetes mortality.

Using WHtR as the independent variable, restricted cubic spline (RCS) curves and Cox regression analyses were performed for cause-specific mortality from cardiovascular disease, cancer, cerebrovascular disease, and diabetes, with adjustments for relevant confounders. RCS plots revealed J-shaped nonlinear relationships (*p* < 0.001) between WHtR and mortality risks for cardiovascular disease, cancer, and diabetes, characterized by initially flat or slightly decreasing curves followed by steep increases, all with inflection points at 0.58. The RCS curve for cerebrovascular disease mortality exhibited a U-shaped pattern but lacked statistical significance (*p* = 0.61) (Figure 4). Cox regression analysis demonstrated that for cardiovascular disease, the lowest mortality risk occurred in Q3 (HR = 0.922, 95% CI: 0.919–0.926), while the highest risk was observed in Q5 (HR = 1.695, 95% CI: 1.689–1.701). Each 0.1-unit increase in WHtR elevated cardiovascular mortality risk by 31.9%, with WHtR ≥ 0.58 associated with a 35.5% risk increase. For cancer, the lowest mortality risk was observed in Q2 (HR = 0.806, 95% CI: 0.803–0.809), while Q5 showed the highest risk (HR = 1.088, 95% CI: 1.085–1.092). Each 0.1-unit WHtR increase raised cancer mortality risk by 6.1%, with WHtR ≥ 0.58 corresponding to a 4.5% risk increase. In diabetes-related mortality, the unadjusted model showed progressively increasing HRs from Q1 to Q5, with Q5 reaching an HR of 7.707 (95% CI: 7.663–7.783) compared to Q1. After adjusting for confounders, Q5 maintained an elevated HR of 2.662 (95% CI: 2.635–2.688). Each 0.1-unit WHtR increase was associated with a 68.7% higher diabetes mortality risk, with WHtR ≥ 0.58 corresponding to a 69.8% risk increase. Cerebrovascular disease mortality showed no significant trend with WHtR, but similar to other obesity-related conditions, WHtR ≥ 0.58 was associated with a 10.0% risk increase (Table 3).

**Table 3:** Adjusted HR (95%CI) of waist-to-height ratio with obesity-related mortality.

Obesity-related mortality	Crude model^a^	Adjusted model^b^	*P-*value
CVD mortality			
Per 0.1	1.595 (1.593–1.596)	1.319 (1.319–1.321)	<0.001
Q1 (<0.50)	1 (Reference)	1 (Reference)	
Q2 (0.50–0.55)	1.982 (1.974–1.989)	1.041 (1.037–1.045)	<0.001
Q3 (0.55–0.60)	2.471 (2.462–2.480)	0.922 (0.919–0.926)	<0.001
Q3 (0.60–0.66)	3.984 (3.970–3.998)	1.194 (1.189–1.198)	<0.001
Q5 (≥0.66)	5.023 (5.066–5.041)	1.695 (1.689–1.701)	<0.001
WhtR ≥ 0.58	2.493 (2.488–2.498)	1.355 (1.353–1.358)	<0.001
Cancer mortality			
Per 0.1	1.327 (1.326–1.328)	1.061 (1.059–1.062)	<0.001
Q1 (<0.50)	1 (Reference)	1 (Reference)	
Q2 (0.50–0.55)	1.320 (1.316–1.324)	0.806 (0.803–0.809)	<0.001
Q3 (0.55–0.60)	1.845 (1.839–1.851)	0.846 (0.843–0.848)	<0.001
Q3 (0.60–0.66)	2.276 (2.269–2.283)	0.872 (0.869–0.875)	<0.001
Q5 (≥0.66)	2.554 (2.546–2.561)	1.088 (1.085–1.092)	<0.001
WhtR ≥ 0.58	1.714 (1.711–1.718)	1.045 (1.043–1.047)	<0.001
Cerebrovascular mortality			
Per 0.1	1.372 (1.369–1.375)	1.029 (1.027–1.032)	<0.001
Q1 (<0.50)	1 (Reference)	1 (Reference)	
Q2 (0.50–0.55)	2.391 (2.371–2.410)	1.168 (1.158–1.177)	<0.001
Q3 (0.55–0.60)	2.175 (2.157–2.193)	0.738 (0.732–0.744)	<0.001
Q3 (0.60–0.66)	4.041 (4.010–4.073)	1.105 (1.096–1.113)	<0.001
Q5 (≥0.66)	3.344 (3.317–3.370)	1.052 (1.044–1.061)	<0.001
WhtR ≥ 0.58	2.054 (2.046–2.063)	1.100 (1.096–1.105)	<0.001
Diabetes mortality			
Per 0.1	1.959 (1.955–1.963)	1.687 (1.682–1.691)	<0.001
Q1 (<0.50)	1 (Reference)	1 (Reference)	
Q2 (0.50–0.55)	2.167 (2.144–2.192)	1.277 (1.263–1.292)	<0.001
Q3 (0.55–0.60)	2.385 (2.360–2.411)	1.011 (1.000–1.022)	<0.001
Q3 (0.60–0.66)	3.838 (3.799–3.879)	1.296 (1.283–1.310)	<0.001
Q5 (≥0.66)	7.707 (7.663–7.783)	2.662 (2.635–2.688)	<0.001
WhtR ≥ 0.58	3.173 (3.156–3.190)	1.698 (1.688–1.707)	<0.001

HR, hazard ratio; Q, quantile.

a Unadjusted.

b Adjusted for age, sex, race and ethnicity, education, income, smoking, drinking, family history of cardiovascular disease, and family history of diabetes.

### 3.4 Subgroup analysis

**Figure 5:**
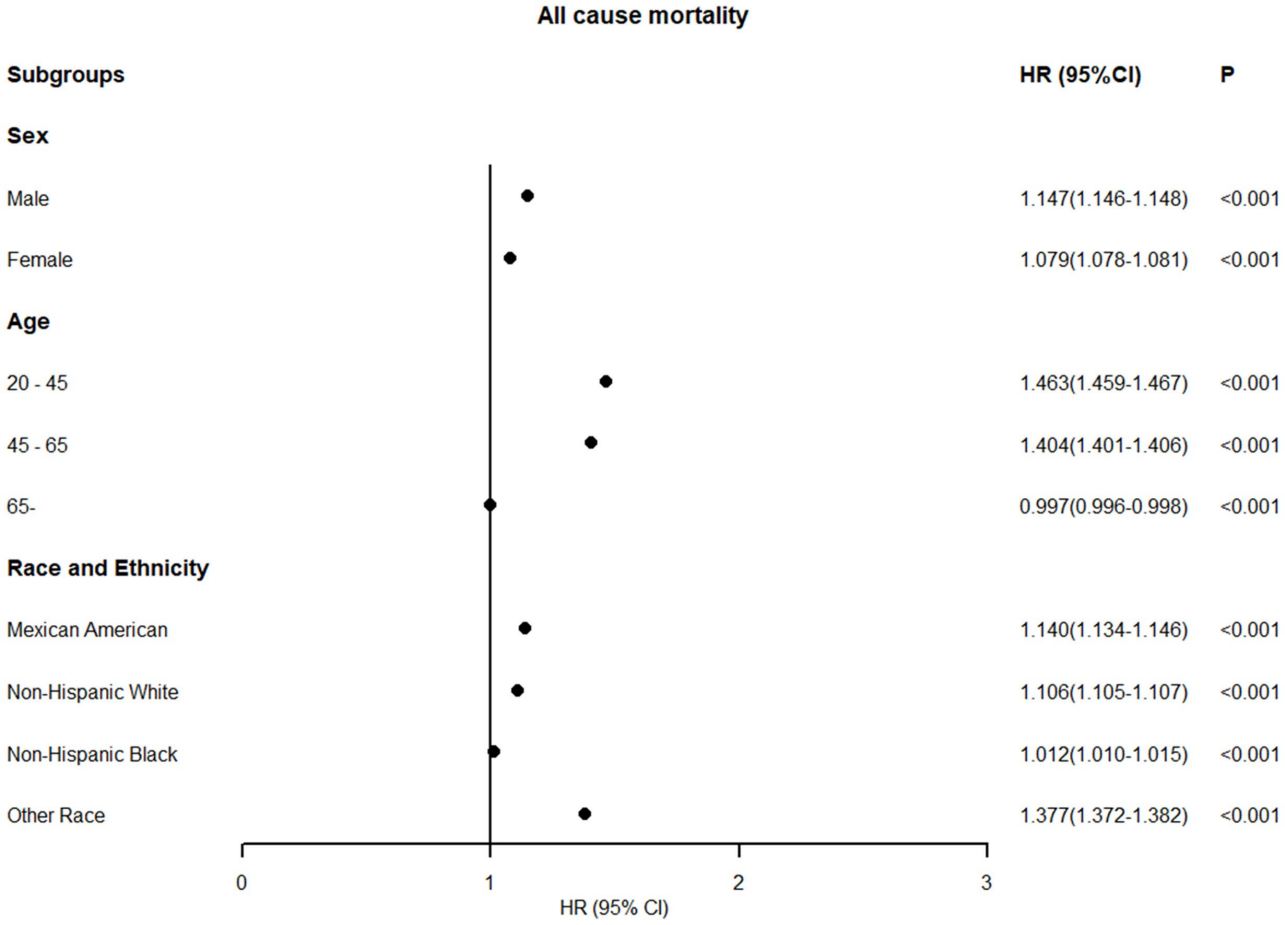
Association between WHtR≥0.58 and all-cause mortality in US adults across different populations (fully adjusted model).

**Figure 6:**
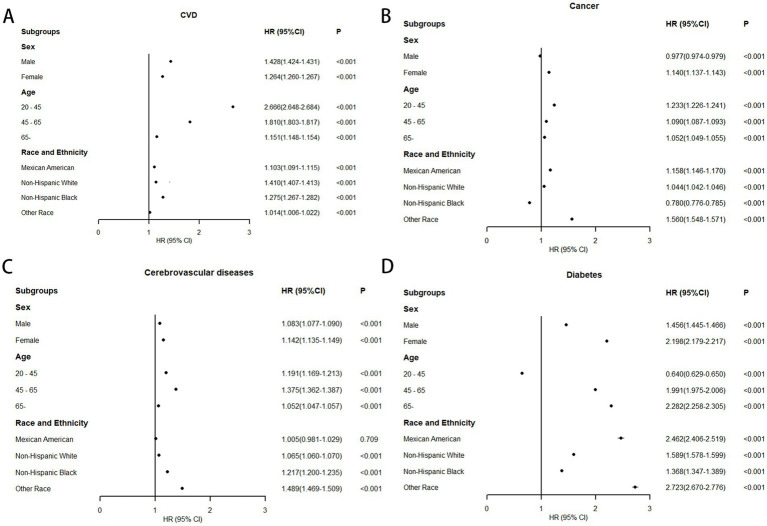
Association between WHtR≥0.58 and obesity-related mortality in US adults across different populations (fully adjusted model). **(A)** Association between WHtR≥0.58 and cardiovascular diseases mortality; **(B)** Association between WHtR≥0.58 and cancer mortality; **(C)** Association between WHtR≥0.58 and cerebrovascular diseases mortality; **(D)** Association between WHtR≥0.58 and diabetes mortality.

Subgroup analyses stratified by sex, age, and ethnicity were conducted using 0.58 as the WHtR cutoff, with individuals having WHtR < 0.58 as the reference group. In all-cause mortality analysis, only older adults (age ≥ 65 years) showed a slight risk reduction (HR = 0.997, 95% CI: 0.996–0.998) when WHtR exceeded 0.58 (Figure 5). For obesity-related disease mortality, only three subgroups demonstrated risk reductions with WHtR > 0.58: cancer mortality in men (HR = 0.977, 95% CI: 0.974–0.979), diabetes mortality in younger adults (HR = 0.640, 95% CI: 0.629–0.650), and cancer mortality in non-Hispanic Black people (HR = 0.780, 95% CI: 0.776–0.785). Moreover, in the subgroup analysis, it is noteworthy that among individuals with a waist-to-height ratio of ≥0.58, the diabetes mortality for females, the elderly, and Mexican Americans, as well as the cardiovascular mortality risk for the young, more than doubled. Specifically, diabetes mortality increased by 119.8% for females, 128.2% for the elderly, and 146.2% for Mexican Americans, while cardiovascular mortality risk rose by 166.6% for the young (Figure 6).

### 3.5 Sensitivity analysis

We conducted sensitivity analysis to verify the stability and extrapolation of the results. Even after excluding participants with less than 2 years of follow-up or those who died from accidents, the results remained significant (Supplementary Tables 1, 2).

## 4 Discussion

This cohort study aimed to investigate the relationship between WHtR and mortality among U. S. adults from 1999 to 2018, providing a scientific and practical anthropometric standard for diverse populations. Baseline data revealed that among 47,741 participants, obesity-related deaths accounted for over half of all mortality during follow-up. The mean WHtR was 0.58, exceeding the central obesity threshold defined by the European Association for the Study of Obesity, with women consistently exhibiting higher WHtR values than men across all age groups. To our knowledge, this represents the first comprehensive investigation of WHtR’s association with all-cause and obesity-related disease mortality in U. S. adults.

Elevated WHtR typically indicates central obesity, reflecting excessive visceral adipose tissue accumulation. Current evidence suggests visceral fat is more metabolically detrimental than other adipose deposits and is independently associated with all-cause mortality and obesity-related disease mortality (16–20). A CT-based study of 2,720 participants demonstrated that increased visceral fat area independently predicts long-term mortality risk (21), establishing visceral fat as the primary pathogenic factor in obesity-related diseases, whereas BMI fails to accurately reflect its distribution (22). A 16.9-year follow-up study identified significant associations between elevated visceral fat and both all-cause mortality (HR 1.39, 95% CI: 1.11–1.75) and obesity-related mortality (HR 1.39, 95% CI: 1.04–1.85), with waist circumference serving as a key mediator in the visceral fat-mortality relationship (23). Visceral fat accumulation, recognized as a marker of systemic lipid metabolism dysfunction, is closely linked to insulin resistance, cardiovascular diseases, and endocrine-metabolic disorders, though waist circumference’s correlation with visceral fat exhibits substantial gender and ethnic variability (24). Emerging evidence positions WHtR as a practical anthropometric indicator for objectively assessing visceral adiposity. However, research on WHtR’s association with obesity-related mortality remains limited, with most studies treating WHtR solely as a categorical or continuous variable and concluding that elevated WHtR correlates with increased mortality risk (25). Existing investigations predominantly focus on cardiovascular outcomes, lacking comprehensive analysis of obesity-related diseases. Establishing simple anthropometric criteria to quantify mortality risk associated with central obesity and identify its inflection points represents a critical research priority.

In contrast to previous studies, we expanded the study population to US adults from 1999 to 2018 and extended follow-up through December 31, 2019, with cardiovascular disease, cancer, cerebrovascular disease, and diabetes-related deaths considered obesity-associated (2). Beyond analyzing waist-to-height ratio as quintiles and continuous variables, we identified 0.58 as the threshold through RCS plots to dichotomize waist-to-height ratio. For all-cause mortality, a U-shaped relationship emerged, with both waist-to-height ratio <0.5 and >0.66 increasing mortality risk. Overall, higher waist-to-height ratio demonstrated greater harm, showing an 11.2% elevated mortality risk when exceeding 0.58. Regarding obesity-related diseases, J-shaped curves characterized waist-to-height ratio associations with cardiovascular disease, cancer, and diabetes mortality, while no clear trend emerged for cerebrovascular mortality. An elevated WHtR (≥0.58) was associated with increased mortality risks for: cardiovascular disease (35.5%), cancer (4.5%), cerebrovascular disease (10.0%), and diabetes (69.8%). The 0.58 threshold remained stable for elevated all-cause and obesity-related mortality across sex-, age-, and race-stratified analyses, except for inverse trends in all-cause mortality among older adults (≥65 years) and cancer mortality in men. Notably, young adults have significantly higher cardiovascular mortality at elevated WHtR, which may be attributable to unmeasured lifestyle factors and genetic determinants. Compared with other age groups, high WHtR in younger individuals demonstrates closer links to adverse lifestyle behaviors. For instance, they exhibit higher propensity for calorie-dense, high-fat, and high-sugar diets coupled with physical inactivity—behavioral patterns that substantially elevate cardiovascular mortality risk. Moreover, young adults with familial cardiovascular disease histories may carry susceptibility genes affecting vascular structure and function (e.g., increased arterial wall fragility or accelerated atherogenesis). Critically, adverse lifestyles can synergistically amplify these genetic risks (26). Specifically, young carriers of susceptibility alleles with high WHtR may experience premature atherosclerotic plaque formation and progression under lifestyle mediation, culminating in early cardiovascular events (e.g., myocardial infarction) and consequent mortality elevation. Additionally, inverse patterns emerged for cancer mortality in non-Hispanic Black people and diabetes mortality in younger adults (<45 years), potentially attributable to lower cancer prevalence among non-Hispanic Black people (27). Regarding diabetes mortality in young adults, On one hand, compared with other populations, young adults have lower diabetes prevalence and fewer diabetes-attributable deaths, potentially limiting the representativeness of these findings. On the other hand, diabetes-related mortality in young adults is more likely associated with type 1 diabetes or genetic defects (28, 29), which may reduce the impact of obesity-related factors. Therefore, the relationship between WHtR and diabetes mortality in young adults requires careful interpretation.

In this cohort study, we investigated the relationship between body composition management and mortality risk through the pathway from waist-to-height ratio to central obesity and visceral adiposity. The study revealed that women and older adults exhibited relatively higher waist-to-height ratios, consistent with previous epidemiological findings, potentially attributable to sex hormone-mediated gender differences and age-related declines in metabolic function of adipose tissue cells (30–32). For all-cause mortality, a U-shaped curve demonstrated that moderate visceral fat levels confer health benefits, aligning with prior research using body roundness index (33). Visceral fat contributes to essential metabolic processes, including energy storage and adipokine secretion (34, 35). When maintained within normal ranges, these physiological functions support systemic health. Individuals with insufficient visceral fat face elevated mortality risks due to compromised energy reserves and immune dysfunction from malnutrition, chronic wasting conditions (e.g., cancer cachexia), or extreme emaciation (36), particularly evident in adults ≥65 years who exhibit poorer nutritional status, where waist-to-height ratio may partially reflect nutritional adequacy. Conversely, excessive visceral fat promotes chronic low-grade inflammation through increased pro-inflammatory adipokine production, impairing insulin sensitivity, accelerating atherosclerosis, and elevating risks of cardiovascular disease, type 2 diabetes, and specific cancers (37–39). The resultant increased incidence of these chronic conditions and complications drives significant rises in all-cause mortality. Regarding obesity-related diseases, elevated waist-to-height ratio demonstrated stronger detrimental effects on cardiovascular and diabetes mortality, consistent with existing literature (16, 20, 23). The weaker association with cancer mortality and observed male obesity paradox may reflect our inclusion of all cancer types rather than obesity-specific malignancies, coupled with hormone-mediated amplification of obesity’s oncogenic effects in females (40) (e.g., ovarian and breast cancers). For cerebrovascular mortality, while high waist-to-height ratio (≥0.58) showed adverse effects, the overall association lacked clear trend consistency. Previous research in this domain remains limited, predominantly focusing on cerebral small vessel diseases including lacunar infarcts, silent brain infarctions, and cerebral microbleeds (41, 42). A cross-sectional study evaluating cerebral vasculopathy risks via visceral-to-subcutaneous fat ratio (VS ratio) reported that each 0.1-unit VS increase elevated risks of cerebral ischemia (OR 1.05[95%CI, 1.01–1.10]), cerebral artery stenosis/occlusion (OR 1.14[95%CI, 1.03–1.25]), and carotid plaque (OR 1.09[95%CI, 1.05–1.13]) (43), underscoring the necessity for comprehensive anthropometric measures and advanced diagnostics to better characterize cerebrovascular impacts of adiposity distribution. The waist-to-height ratio’s mortality associations remained robust across age, gender, and ethnic subgroups. Our analysis identified high-risk populations: younger individuals showed greater waist-to-height ratio-associated cardiovascular mortality, potentially explaining elevated sudden death rates in obese youth (44, 45), while older adults, women, and Mexican Americans exhibited heightened diabetes mortality sensitivity to waist-to-height ratio increases, indicating stricter body composition monitoring needs.

This study yielded three principal findings: First, the U-shaped relationship between waist-to-height ratio and all-cause mortality identified 0.58 as the inflection point, slightly below the US adult average, suggesting no universally optimal body composition exists—maintaining moderate ranges may better adapt to modern healthcare environments. Second, in obesity-related diseases, a waist-to-height ratio of ≥0.58 is a reliable indicator for increased mortality risk. Finally, waist-to-height ratio warrants particular consideration for diabetes management in elderly and female populations.

This study possesses several strengths, including long-term follow-up, a nationally representative sample, and comprehensive adjustment for confounding factors. Additionally, it provides more precise cutoff values for waist-to-height ratio mortality associations validated across sex, age, and racial subgroups. Several limitations should be acknowledged: First, the inclusion of all cancer-related mortality regardless of obesity association in our dataset may have diminished waist-to-height ratio’s predictive accuracy for cancer mortality. Second, the indeterminate relationship between waist-to-height ratio and cerebrovascular mortality requires further investigation. Third, Given the extended follow-up duration, our findings may be subject to time-dependent confounding and reverse causation issues, particularly concerning the relationship between low WHtR and mortality in the elderly population. Finally, as an anthropometric indicator reflecting visceral adiposity, waist-to-height ratio fails to capture subcutaneous adipose tissue distribution, limiting its capacity to characterize overall fat compartmentalization.

## 5 Conclusion

In this national cohort study, waist-to-height ratio shows a U-shaped relation to all-cause mortality. It also offers a stable cut-off (0.58) for obesity - related disease mortality, unaffected by age, sex, or race. This supports waist-to-height ratio as a simple, non-invasive measure for visceral fat assessment and high-risk population screening, giving the public a precise anthropometric indicator for body management. However, more clinical research is needed to boost its chances of being included in public health policies.

## Data Availability

The original contributions presented in the study are included in the article/Supplementary material, further inquiries can be directed to the corresponding authors.

## References

[1] Islam A Sultana H Nazmul Hassan Refat M Farhana Z Abdulbasah Kamil A Meshbahur Rahman M. The global burden of overweight-obesity and its association with economic status, benefiting from STEPs survey of WHO member states: a meta-analysis. Prev Med Rep. (2024) 46:102882. doi: 10.1016/j.pmedr.2024.102882, PMID: 39290257 PMC11406007

[2] Ard J. Obesity in the US: what is the best role for primary care? BMJ. (2015) 350:g7846. doi: 10.1136/bmj.g7846, PMID: 25656059

[3] Wen X Zhang B Wu B Xiao H Li Z Li R. Signaling pathways in obesity: mechanisms and therapeutic interventions. Signal Transduct Target Ther. (2022) 7:298. doi: 10.1038/s41392-022-01149-x, PMID: 36031641 PMC9420733

[4] Barrea L Boschetti M Gangitano E Guglielmi V Verde L Muscogiuri G. Long-term efficacy and safety of nutritional and pharmacological strategies for obesity. Curr Obes Rep. (2025) 14:1. doi: 10.1007/s13679-024-00602-y, PMID: 39753703

[5] Simati S Kokkinos A Dalamaga M Argyrakopoulou G. Obesity paradox: fact or fiction? Curr Obes Rep. (2023) 12:75–85. doi: 10.1007/s13679-023-00497-1 36808566

[6] Neeland IJ Ross R Després JP Matsuzawa Y Yamashita S Shai I. Visceral and ectopic fat, atherosclerosis, and cardiometabolic disease: a position statement. Lancet Diab Endocrinol. (2019) 7:715–25. doi: 10.1016/S2213-8587(19)30084-1, PMID: 31301983

[7] Jayedi A Soltani S Zargar MS Khan TA Shab-Bidar S. Central fatness and risk of all cause mortality: systematic review and dose-response meta-analysis of 72 prospective cohort studies. BMJ (Clinical research ed). (2020) 370:m3324. doi: 10.1136/bmj.m3324 PMC7509947 32967840

[8] Rubino F Cummings DE Eckel RH Cohen RV Wilding JPH Brown WA. Definition and diagnostic criteria of clinical obesity. Lancet Diab Endocrinol. (2025) 13:221–62. doi: 10.1016/S2213-8587(24)00316-4, PMID: 39824205 PMC11870235

[9] Busetto L Dicker D Frühbeck G Halford JCG Sbraccia P Yumuk V. A new framework for the diagnosis, staging and management of obesity in adults. Nat Med. (2024) 30:2395–9. doi: 10.1038/s41591-024-03095-3, PMID: 38969880

[10] Butt JH Petrie MC Jhund PS Sattar N Desai AS Køber L. Anthropometric measures and adverse outcomes in heart failure with reduced ejection fraction: revisiting the obesity paradox. Eur Heart J. (2023) 44:1136–53. doi: 10.1093/eurheartj/ehad083 36944496 PMC10111968

[11] Ashwell M Gunn P Gibson S. Waist-to-height ratio is a better screening tool than waist circumference and BMI for adult cardiometabolic risk factors: systematic review and meta-analysis. Obes Rev. (2012) 13:275–86. doi: 10.1111/j.1467-789X.2011.00952.x, PMID: 22106927

[12] Zong X Kelishadi R Hong YM Schwandt P Matsha TE Mill JG. Establishing international optimal cut-offs of waist-to-height ratio for predicting cardiometabolic risk in children and adolescents aged 6-18 years. BMC Med. (2023) 21:442. doi: 10.1186/s12916-023-03169-y, PMID: 37968681 PMC10647138

[13] Jia AH Xu SY Ming J Zhou J Zhang WC Hao PR. The optimal cutoff value of waist-to-height ratio in Chinese: based on cardiovascular risk and metabolic disease. Zhonghua Nei Ke Za Zhi. (2017) 56:822–6. doi: 10.3760/cma.j.issn.0578-1426.2017.11.009, PMID: 29136711

[14] Choi DH Hur YI Kang JH Kim K Cho YG Hong SM. Usefulness of the waist circumference-to-height ratio in screening for obesity and metabolic syndrome among Korean children and adolescents: Korea National Health and nutrition examination survey, 2010-2014. Nutrients. (2017) 9:256. doi: 10.3390/nu9030256, PMID: 28287410 PMC5372919

[15] Louie JCY Wall-Medrano A. Editorial: waist-to-height ratio is a simple tool for assessing central obesity and consequent health risk. Front Nutr. (2023) 10:1277610. doi: 10.3389/fnut.2023.1277610, PMID: 37823085 PMC10562721

[16] Li L Li W Xu D He H Yang W Guo H. Association between visceral fat area and Cancer prognosis: a population-based multicenter prospective study. Am J Clin Nutr. (2023) 118:507–17. doi: 10.1016/j.ajcnut.2023.07.001, PMID: 37422158

[17] Feng H Wang X Zhao T Mao L Hui Y Fan X. Myopenic obesity determined by visceral fat area strongly predicts long-term mortality in cirrhosis. Clin Nutr (Edinburgh, Scotland). (2021) 40:1983–9. doi: 10.1016/j.clnu.2020.09.016, PMID: 32977996

[18] Bae J Ju JW Lee S Nam K Kim TK Jeon Y. Association between abdominal fat and mortality in patients undergoing cardiovascular surgery. Ann Thorac Surg. (2022) 113:1506–13. doi: 10.1016/j.athoracsur.2021.05.049, PMID: 34116000

[19] Saad RK Ghezzawi M Horanieh R Khamis AM Saunders KH Batsis JA. Abdominal visceral adipose tissue and all-cause mortality: a systematic review. Front Endocrinol. (2022) 13:922931. doi: 10.3389/fendo.2022.922931, PMID: 36082075 PMC9446237

[20] Zhu Y Zou H Guo Y Luo P Meng X Li D. Associations between metabolic score for visceral fat and the risk of cardiovascular disease and all-cause mortality among populations with different glucose tolerance statuses. Diabetes Res Clin Pract. (2023) 203:110842. doi: 10.1016/j.diabres.2023.110842 37495020

[21] Lee JH Choi SH Jung KJ Goo JM Yoon SH. High visceral fat attenuation and long-term mortality in a health check-up population. J Cachexia Sarcopenia Muscle. (2023) 14:1495–507. doi: 10.1002/jcsm.13226, PMID: 37016984 PMC10235877

[22] Mitsushio K Baden MY Kagisaki T Kato S Niki A Takayama R. Interrelationships among accumulations of intra-and Periorgan fats, visceral fat, and subcutaneous fat. Diabetes. (2024) 73:1122–6. doi: 10.2337/db24-0035, PMID: 38656942 PMC11189825

[23] McNeely MJ Shofer JB Leonetti DL Fujimoto WY Boyko EJ. Associations among visceral fat, all-cause mortality, and obesity-related mortality in Japanese Americans. Diabetes Care. (2012) 35:296–8. doi: 10.2337/dc11-1193, PMID: 22190675 PMC3263911

[24] Bays HE. Evaluation and practical Management of Increased Visceral fat: should cardiologists lose sleep over it? J Am Coll Cardiol. (2022) 79:1266–9. doi: 10.1016/j.jacc.2022.01.039 35361349

[25] Abdi Dezfouli R Mohammadian Khonsari N Hosseinpour A Asadi S Ejtahed HS Qorbani M. Waist to height ratio as a simple tool for predicting mortality: a systematic review and meta-analysis. Int J Obes. (2023) 47:1286–301. doi: 10.1038/s41366-023-01388-0 37770574

[26] Khera AV Emdin CA Drake I Natarajan P Bick AG Cook NR. Genetic risk, adherence to a healthy lifestyle, and coronary disease. N Engl J Med. (2016) 375:2349–58. doi: 10.1056/NEJMoa1605086, PMID: 27959714 PMC5338864

[27] Williams PA Zaidi SK Ramian H Sengupta R. AACR Cancer disparities Progress report 2024: achieving the bold vision of health equity. Cancer Epidemiol Biomarkers Prev. (2024) 33:870–3. doi: 10.1158/1055-9965.EPI-24-0658, PMID: 38748491

[28] Murtaza G Riaz S Zafar M Ahsan Raza M Kaleem I Imran H. Examining the growing challenge: prevalence of diabetes in young adults (review). Med Int. (2025) 5:2. doi: 10.3892/mi.2024.201, PMID: 39563945 PMC11571047

[29] Lawrence JM Slezak JM Quesenberry C Li X Yu L Rewers M. Incidence and predictors of type 1 diabetes among younger adults aged 20-45 years: the diabetes in young adults (DiYA) study. Diabetes Res Clin Pract. (2021) 171:108624. doi: 10.1016/j.diabres.2020.108624, PMID: 33338552 PMC10116767

[30] Lovejoy JC Sainsbury A. Sex differences in obesity and the regulation of energy homeostasis. Obes Rev. (2009) 10:154–67. doi: 10.1111/j.1467-789X.2008.00529.x, PMID: 19021872

[31] Mahboobifard F Pourgholami MH Jorjani M Dargahi L Amiri M Sadeghi S. Estrogen as a key regulator of energy homeostasis and metabolic health. Biomed Pharmacother. (2022) 156:113808. doi: 10.1016/j.biopha.2022.113808, PMID: 36252357

[32] Liu Z Wu KKL Jiang X Xu A Cheng KKY. The role of adipose tissue senescence in obesity-and ageing-related metabolic disorders. Clin Sci (London, England: 1979). (2020) 134:315–30. doi: 10.1042/CS20190966, PMID: 31998947

[33] Zhang X Ma N Lin Q Chen K Zheng F Wu J. Body roundness index and all-cause mortality among US adults. JAMA Netw Open. (2024) 7:e2415051. doi: 10.1001/jamanetworkopen.2024.15051, PMID: 38837158 PMC11154161

[34] Tchernof A Després JP. Pathophysiology of human visceral obesity: an update. Physiol Rev. (2013) 93:359–404. doi: 10.1152/physrev.00033.2011, PMID: 23303913

[35] Sakers A De Siqueira MK Seale P Villanueva CJ. Adipose-tissue plasticity in health and disease. Cell. (2022) 185:419–46. doi: 10.1016/j.cell.2021.12.016, PMID: 35120662 PMC11152570

[36] Wang H Zhao T Guo G Yang W Zhang X Yang F. Global leadership initiative on malnutrition-defined malnutrition coexisting with visceral adiposity predicted worse long-term all-cause mortality among inpatients with decompensated cirrhosis. Nutr Diabetes. (2024) 14:76. doi: 10.1038/s41387-024-00336-9, PMID: 39333477 PMC11436742

[37] Kolb H. Obese visceral fat tissue inflammation: from protective to detrimental? BMC Med. (2022) 20:494. doi: 10.1186/s12916-022-02672-y, PMID: 36575472 PMC9795790

[38] Li H Meng Y He S Tan X Zhang Y Zhang X. Macrophages, chronic inflammation, and insulin resistance. Cells. (2022) 11:3001. doi: 10.3390/cells11193001, PMID: 36230963 PMC9562180

[39] Stefan N. Causes, consequences, and treatment of metabolically unhealthy fat distribution. The lancet Diabetes & endocrinology. (2020) 8:616–27. doi: 10.1016/S2213-8587(20)30110-8, PMID: 32559477

[40] Avgerinos KI Spyrou N Mantzoros CS Dalamaga M. Obesity and cancer risk: emerging biological mechanisms and perspectives. Metab Clin Exp. (2019) 92:121–35. doi: 10.1016/j.metabol.2018.11.001, PMID: 30445141

[41] Nam KW Kwon HM Jeong HY Park JH Kwon H. Association of Body Shape Index with cerebral small vessel disease. Obes Facts. (2023) 16:204–11. doi: 10.1159/000528701, PMID: 36535265 PMC10028365

[42] Yamashiro K Tanaka R Tanaka Y Miyamoto N Shimada Y Ueno Y. Visceral fat accumulation is associated with cerebral small vessel disease. Eur J Neurol. (2014) 21:667–73. doi: 10.1111/ene.12374, PMID: 24495037

[43] Higuchi S Kabeya Y Kato K. Visceral-to-subcutaneous fat ratio is independently related to small and large cerebrovascular lesions even in healthy subjects. Atherosclerosis. (2017) 259:41–5. doi: 10.1016/j.atherosclerosis.2017.03.001, PMID: 28285092

[44] Andersson C Vasan RS. Epidemiology of cardiovascular disease in young individuals. Nat Rev Cardiol. (2018) 15:230–40. doi: 10.1038/nrcardio.2017.154, PMID: 29022571

[45] Holmstrom L Junttila J Chugh SS. Sudden death in obesity: mechanisms and management. J Am Coll Cardiol. (2024) 84:2308–24. doi: 10.1016/j.jacc.2024.09.016, PMID: 39503654

