# Supplementary material for: Association of waist-to-height ratio with all-cause and obesity-related mortality in adults: a prospective cohort study
Source: Front Nutr. 2025 Aug 11;12:1614347. doi: 10.3389/fnut.2025.1614347 (PMC12375492; doi:10.3389/fnut.2025.1614347)
Supplement: Supplementary file 1 [file Table_1.DOCX]

eTable1. Adjusted HR(95%CI) of Waist-to-Height Ratio with all-cause mortality After excluding participants with less than 2 years of follow - up or those who died from accidents

|  | Crude model^a^ | Adjusted model^b^ | P-value |
| --- | --- | --- | --- |
| All cause mortality |  |  |  |
| Per 0.1 | 1.448(1.448-1.449) | 1.131(1.131-1.132) | ＜0.001 |
| Q1(＜0.50) | 1(Reference) | 1(Reference) |  |
| Q2(0.50-0.55) | 1.557(1.555-1.560) | 0.871(0.869-0.872) | ＜0.001 |
| Q3(0.55-0.60) | 1.880(1.877-1.884) | 0.759(0.758-0.761) | ＜0.001 |
| Q3(0.60-0.66) | 2.771(2.766-2.775) | 0.898(0.897-0.900) | ＜0.001 |
| Q5(≥0.66) | 3.230(3.224-3.235) | 1.153(1.151-1.155) | ＜0.001 |
| WhtR≥0.58 | 1.998(1.996-2.000) | 1.109(1.108-1.110) | ＜0.001 |

Abbreviations: HR, hazard ratio; Q, quantile.

^a^ unadjusted.

^b^ Adjusted for age, sex, race and ethnicity, education, income, smoking, drinking, family history of cardiovascular disease, and family history of diabetes.

eTable2. Adjusted HR(95%CI) of Waist-to-Height Ratio with Obesity-related Mortality After excluding participants with less than 2 years of follow - up or those who died from accidents

|  | Crude model^a^ | Adjusted model^b^ | P-value |
| --- | --- | --- | --- |
| CVD mortality |  |  |  |
| Per 0.1 | 1.617(1.616-1.618) | 1.331(1.329-1.332) | ＜0.001 |
| Q1(＜0.50) | 1(Reference) | 1(Reference) |  |
| Q2(0.50-0.55) | 2.187(2.178-2.196) | 1.135(1.130-1.140) | ＜0.001 |
| Q3(0.55-0.60) | 2.723(2.715-2.734) | 1.001(0.997-1.005)^c^ | ＜0.001 |
| Q3(0.60-0.66) | 4.306(4.290-4.323) | 1.264(1.259-1.269) | ＜0.001 |
| Q5(≥0.66) | 5.563(5.542-5.584) | 1.838(1.831-1.845) | ＜0.001 |
| WhtR≥0.58 | 2.540(5.535-2.545) | 1.363(1.360-1.366) | ＜0.001 |
| Cancer mortality |  |  |  |
| Per 0.1 | 1.338(1.337-1.339) | 1.065(1.064-1.066) | ＜0.001 |
| Q1(＜0.50) | 1(Reference) | 1(Reference) |  |
| Q2(0.50-0.55) | 1.349(1.345-1.354) | 0.827(0.824-0.830) | ＜0.001 |
| Q3(0.55-0.60) | 1.806(1.800-1.812) | 0.832(0.829-0.835) | ＜0.001 |
| Q3(0.60-0.66) | 2.397(2.389-2.405) | 0.920(0.917-0.924) | ＜0.001 |
| Q5(≥0.66) | 2.618(2.609-2.626) | 1.115(1.111-1.119) | ＜0.001 |
| WhtR≥0.58 | 1.773(1.769-1.776) | 1.078(1.076-1.080) | ＜0.001 |
| Cerebrovascular mortality |  |  |  |
| Per 0.1 | 1.442(1.439-1.445) | 1.095(1.092-1.098) | ＜0.001 |
| Q1(＜0.50) | 1(Reference) | 1(Reference) |  |
| Q2(0.50-0.55) | 2.765(2.740-2.790) | 1.323(1.311-1.335) | ＜0.001 |
| Q3(0.55-0.60) | 2.646(2.623-2.671) | 0.865(0.857-0.873) | ＜0.001 |
| Q3(0.60-0.66) | 4.953(4.910-4.995) | 1.302(1.291-1.313) | ＜0.001 |
| Q5(≥0.66) | 4.254(4.217-4.292) | 1.278(1.266-1.289) | ＜0.001 |
| WhtR≥0.58 | 2.234(2.224-2.244) | 1.169(1.163-1.174) | ＜0.001 |
| Diabetes mortality |  |  |  |
| Per 0.1 | 1.914(1.910-1.918) | 1.629(1.624-1.633) | ＜0.001 |
| Q1(＜0.50) | 1(Reference) | 1(Reference) |  |
| Q2(0.50-0.55) | 2.132(2.109-2.156) | 1.262(1.248-1.276) | ＜0.001 |
| Q3(0.55-0.60) | 2.338(2.313-2.364) | 0.985(0.974-0.996) | ＜0.001 |
| Q3(0.60-0.66) | 3.438(3.402-3.475) | 1.152(1.140-1.165) | ＜0.001 |
| Q5(≥0.66) | 7.151(7.081-7.221) | 2.461(2.436-2.486) | ＜0.001 |
| WhtR≥0.58 | 2.939(2.923-2.955) | 1.562(1.553-1.570) | ＜0.001 |

Abbreviations: HR, hazard ratio; Q, quantile.

^a^ Unadjusted.

^b^ Adjusted for age, sex, race and ethnicity, education, income, smoking, drinking, family history of

cardiovascular disease, and family history of diabetes

^c^ P=0.688
